# Enkephalin as a Pivotal Player in Neuroadaptations Related to Psychostimulant Addiction

**DOI:** 10.3389/fpsyt.2018.00222

**Published:** 2018-05-28

**Authors:** Bethania Mongi-Bragato, María P. Avalos, Andrea S. Guzmán, Flavia A. Bollati, Liliana M. Cancela

**Affiliations:** Instituto de Farmacología Experimental de Córdoba (IFEC-CONICET), Departamento de Farmacología, Facultad de Ciencias Químicas, Universidad Nacional de Córdoba, Córdoba, Argentina

**Keywords:** enkephalin, cocaine, amphetamine, neuroadaptations, addiction, opioid antagonists

## Abstract

Enkephalin expression is high in mesocorticolimbic areas associated with psychostimulant-induced behavioral and neurobiological effects, and may also modulate local neurotransmission in this circuit network. Psychostimulant drugs, like amphetamine and cocaine, significantly increase the content of enkephalin in these brain structures, but we do not yet understand the specific significance of this drug-induced adaptation. In this review, we summarize the neurochemical and molecular mechanism of psychostimulant-induced enkephalin activation in mesocorticolimbic brain areas, and the contribution of this opioid peptide in the pivotal neuroadaptations and long-term behavioral changes underlying psychostimulant addiction. There is evidence suggesting that adaptive changes in enkephalin content in the mesocorticolimbic circuit, induced by acute and chronic psychostimulant administration, may represent a key initial step in the long-term behavioral and neuronal plasticity induced by these drugs.

## Introduction

Psychostimulant addiction is a severe worldwide health problem. The most challenging aspects in its treatment are compulsive drug use and relapse. Currently, there are no effective pharmacotherapies for this disorder. New therapeutic approaches are required based on understanding the neurobiology of drug addiction. Numerous lines of research suggest that exposure to psychostimulant drugs causes neurochemical and molecular adaptations that explain the stability of the behavioral disorders characterizing the addictive state (1, 2). Attention has focused on how the mesocorticolimbic dopamine circuit is affected by drugs of abuse, and particularly on the role of glutamate and dopamine neurotransmission in determining the neuroplastic changes related to psychostimulant addiction (3–6). At molecular level, it has been shown that activation of glutamate and dopamine neurotransmission after repeated psychostimulant administrations, affects intracellular signaling cascades (7–9), alters the expression of membrane receptors (10, 11) and changes gene expression within the neural circuits (12, 13), which leads to sensitization of the drug's behavioral effects (14) and other behavioral alterations observed in addiction, like intense drug craving and relapse (15).

Enkephalin, an opioid peptide derived from proenkephalin (PENK), is widely expressed in the mesocorticolimbic circuit (16) and interacts with glutamate and dopamine in the brain reward structures related to psychostimulant-induced effects. Both delta-opioid (DOPr) and mu-opioid receptors (MOPr) can be activated by enkephalin, and each has its particular pattern of expression within the motivational circuit (17). Although several pharmacological and genetic approaches demonstrate a role of both MOPr and DOPr in psychostimulant-induced behavioral effects, the role of the endogenous opioid peptides in this process has not been fully examined. Previous studies from our lab have demonstrated a long-lasting increase in enkephalin levels within the mesocorticolimbic circuit after psychostimulant administration (18, 19). Enkephalin has also been shown to positively modulate dopamine and glutamate neurotransmission within this circuit (20–25). These data indicate that cocaine-induced enkephalin elevation may drive the neuronal plasticity induced by the drug and the long-term behavioral effects of psychostimulant exposure.

## Activation of the enkephalin system by psychostimulants in the central nervous system

Mesolimbic dopamine activity is directly affected by psychostimulants (26). These drugs bind to monoamine transporters and block reuptake mechanisms (cocaine), or competitively inhibit dopamine uptake and disrupt vesicular storage (amphetamine). The activation of this system is a primary conditioner of their psychomotor stimulant and rewarding effects (27). Acute or chronic administration of psychostimulants also alters, among others, the levels of endogenous opioid peptides, including enkephalin, within areas of the mesocorticolimbic circuit.

Acute cocaine (28, 29) and amphetamine (30–33) elevate PENK mRNA levels in the striatum, and these levels are also increased after chronic cocaine exposure in different dopamine mesolimbic afferents (34, 35, 36). Elevated PENK levels were also observed within the caudate putamen on the second day of binge cocaine administration (37). Following chronic cocaine treatment, no changes were observed in the cortex in PENK mRNA levels (38), prefrontal cortex (39, 40), amygdala (41, 42), hypothalamus, pituitary, central gray and cerebellum, nucleus accumbens or caudate putamen (39). However, PENK mRNA levels were significantly elevated during long-term extinction (10 days) of a cocaine self-administration paradigm in the caudate putamen, nucleus accumbens, piriform cortex and olfactory tubercle regions, and decreased in the central amygdale of rats (43). Similarly, sensitized PENK mRNA expression was observed in the nucleus accumbens and/or caudate putamen in response to an amphetamine challenge following acute (44–47) or chronic (48) amphetamine pretreatment in animals after short-term abstinence from the drug.

Furthermore, data from our lab demonstrate an increase in the levels of met-enkephalin in the nucleus accumbens from rats after acute amphetamine (5 mg/kg i.p.) following 4, but not 7 or 21 days after the last drug injection (18, 49, 50). Met-enkephalin elevation was also observed after 4 days withdrawal period from chronic amphetamine (5 × 2 mg/kg i.p) (49). Interestingly, long-lasting sensitization to amphetamine-induced increases in met-enkephalin levels was evidenced in the same brain area following amphetamine challenge (1 mg/kg i.p.) 21 days after the last acute (5 mg/kg i.p.) administration of the drug (18). Similarly, persistent met-enkephalin immunoreactivity was evidenced in the nucleus accumbens from mice treated chronically with cocaine (9 × 15 mg/kg i.p.) after a long-term abstinence from the drug (12 days after last injection). Met-enkephalin immunoreactivity elevations induced by chronic cocaine is not dependent of cocaine challenge administration (7.5 mg/kg i.p., day 21), as this effect on met-enkephalin immunoreactivity was also observed after saline challenge injection (19).

Altogether these data demonstrate that PENK mRNA levels are increased in specific dopaminergic regions following psychostimulant administration, be the injection acute, chronic or remote.

## Neurochemical and molecular mechanisms in psychostimulant-induced proenkephalin expression

**Figure 1:**
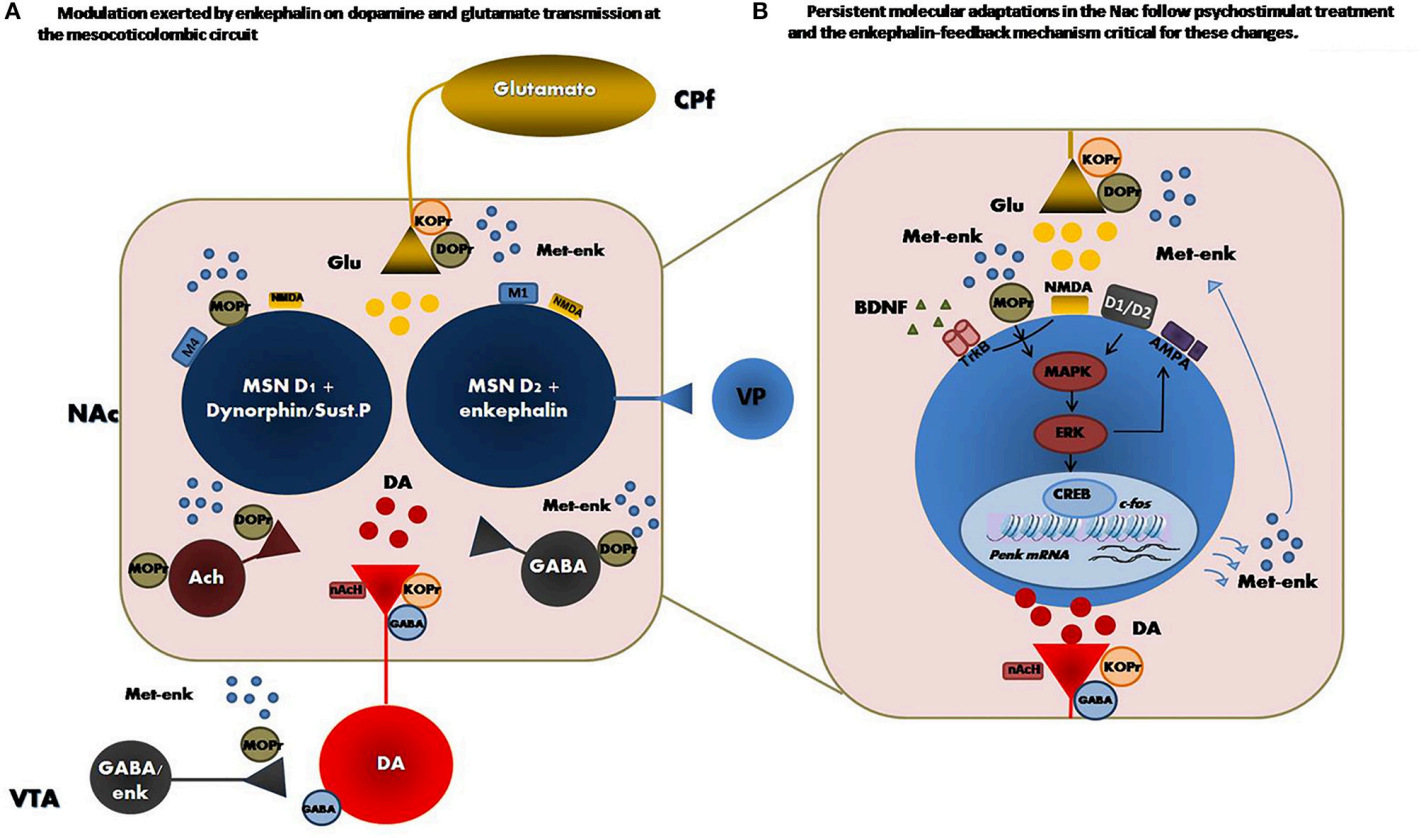
**(Left)** Principal met-enkephalin target nucleus in the mesocorticolimbic circuit. Distribution of opioid receptors within the circuit network is also shown and reveals the modulation exerted by enkephalin on dopamine and glutamate transmission at this level. **(Right)** Persistent adaptations in the enkephalin content followed by psychostimulant treatment and the subsequent activation of MOPr could result in a feedback mechanism critical for the neuronal plasticity induced by these drugs in the NAc. Enkephalin transmission activation promotes the development of psychostimulant-induced long-term neurochemical and molecular changes in the NAc, such as increases in BDNF/TrkB, phospho-ERK2/CREB signaling activation and GluR1 AMPA cell surface expression. VP, ventral pallidum; VTA, ventral tegmentalarea; PfC, Prefrontal cortex; DA, dopamine; Ach, acetylcholine; Glu, glutamate; met-enk, methionine encephalin; GABA, γ-aminobutyric, M1 and M4: muscarinic acetylcholine receptors type 1 and 4, respectively, acetylcholine nAch, nicotinic acetylcholine receptor.

Psychostimulant-induced PENK mRNA expression at striatal level may be the result of multiple neurotransmitter interactions (31, 51, 52). Cocaine and amphetamine stimulate the PENK mRNA expression in striatal neurons (19, 28, 31, 35, 49), which mostly express D2 receptors (53, 54), and also induce prodynorphin and substance P in striatal neurons (31), which mainly express D1 receptors (53, 55). Similarly, the full D1 receptor agonist SKF-82958 induced PENK, prodynorphin and substance P gene expression in both the dorsal and ventral striatum (33). Interestingly, the increase in met-enkephalin induced by amphetamine (50) or PENK mRNA levels stimulated by SKF-82958 in striatal neurons (33) was blocked by the D1 receptor antagonist SCH-23390 (50) and by scopolamine, the muscarinic receptor antagonist (32). Oppositely, the D2 receptor antagonist eticlopride did not affect SKF-82958-induced PENK mRNA expression (33). Similarly, amphetamine-induced met-enkephalin levels was not modified by raclopride, another D2 receptor antagonist (50). Thus, this evidence suggests that D1-mediated induction of PENK may involve trans-synaptic activation of cholinergic neurotransmission. That is, the psychostimulant-induced dopamine elevations stimulates acetylcholine release via a D1-dependent mechanism (56, 57). The acetylcholine released then activates muscarinic M1 receptors (32, 44) and associative signaling pathways in enkephalin-containing neurons thus facilitating PENK mRNA expression (Figure 1). Opioid receptors located at striatal level are also involved in psychostimulant-induced PENK mRNA expression. Selective kappa opioid receptor (KOPr) agonists appear to inhibit psychostimulant-induced alterations in PENK mRNA in the striatum (58), and DOPr antagonists significantly decreased amphetamine-induced mRNA PENK expression (45). In contrast to DOPr's inhibitory effects, MOPr antagonists, alone or combined with amphetamine, increase PENK mRNA levels in the dorsal striatum (45). Opioid receptors thus probably differentially regulate psychostimulant-induced PENK gene expression in the striatum, as a result of the predominantly MOPr expression at D1+ medium spiny neuron vs. D2+ medium spiny neuron and the selective pre-synaptic DOPr location in the local network. Similarly, pre-synaptic KOPr located at striatal dopamine and glutamate nerve terminals could regulates psychostimulant-evoked neurotransmitter release (59) indirectly affecting PENK expression within this brain area.

Glutamate transmission actively regulates PENK gene expression under normal or stimulated conditions (51, 52). However, the precise mechanism by which glutamate participates in psychostimulant-stimulated PENK mRNA expression requires further study. Several reports indicate that cocaine (60, 61) and amphetamine (62–64) administration increases extracellular glutamate levels as well as dopamine levels in the striatum. Also, glutamate tone may be important for amphetamines to stimulate dopamine release from nerve terminals (65, 66, 67, 64). Thus, glutamate transmission could also play a role in regulating the stimulant effect of psychostimulants on PENK mRNA expression. There is evidence from our lab that pretreatment with NMDA receptor antagonists attenuates long-lasting amphetamine-induced PENK mRNA expression and met-enkephalin levels in the nucleus accumbens (18). In addition, there is evidence that glutamate transmission mediated by the AMPA receptor is involved in acute amphetamine-induced PENK levels in the striatum (52). Alternatively, elevated glutamate transmission seems to increase acetylcholine release (68, 69), and this induces acetylcholine-sensitive PENK gene expression, possibly through a NMDA receptor mechanism.

The regulation of PENK in the brain is usually preceded by the induction of AP-1, cAMP response element-binding protein (CREB) and c-Fos (70–74). Dopamine D1 receptor stimulation activates these transcription factors and, if dopamine D2 is also activated, there is a synergistic mechanism (75, 76). This initiates a sequence of molecular steps critically involved in psychostimulant-induced behavioral responses. CREB is the primary regulator of transcriptional activity in accumbal projection neurons and is phosphorylated by protein kinases, including the extracellular signaling-regulated kinase (ERK1/2) (77, 78). Glutamate-stimulated CREB phosphorylation in the striatum is attenuated by the ERK1/2 kinase inhibitor, PD98059 (77–79).

Psychostimulant drugs, which increase dopamine, glutamate and PENK content in mesocorticolimbic brain areas, also up-regulate ERK2/CREB phosphorylation (8, 9, 80). Consistent with this, the inhibition of the ERK2/CREB signaling pathway prevents the increase of psychostimulant-induced PENK mRNA expression (47). This strongly indicates that the long-term increase in met-enkephalin levels, induced by psychostimulants in mesocorticolimbic brain structures, is mediated by a dopamine- and glutamate-dependent mechanism, with the activation of dopamine D1 and glutamate NMDA receptors leading to ERK2/CREB signaling pathway activation in the same brain areas.

## Role of the enkephalinergic system in psychostimulant-induced long-term behavioral effects and associated neuroadaptations

Although enkephalin seems to exert an influence on key areas involved in psychostimulant-induced behavioral effects, the mechanism underlying long-term effects has not yet been fully explained. Pharmacologically, PENK-derived opioid peptides seem to show high affinity for DOPr, but also good affinity for MOPr (17). Furthermore, dopamine release in the nuceleus accumbens appears to be promoted by enkephalin in the ventral tegmental area (20, 81), while MOPr antagonists administered intra-ventral tegmental area cause a decrease in dopamine neurotransmission (82). Pharmacological studies have shown that MOPr and DOPr contribute to increasing dopamine and glutamate release induced by psychostimulants in the nucleus accumbens (83–86). Consistently, pharmacological approaches using MOPr and DOPr antagonists, as well as MOPr knockout mice, demonstrate that the endogenous opioid system is involved in dopamine-related behaviors (87–89). This evidence, together with studies showing that PENK is one of the mediators of the positive reinforcing effects of nicotine, alcohol and marihuana (90–92), suggests that enkephalin may also have a role in psychostimulant-induced behaviors. However, further study is needed to explain the mechanism of its involvement.

### Behavioral sensitization

Repeated intermittent exposure to cocaine steadily increases the locomotor response to the drug (behavioral sensitization) (14), which is mostly coupled to a greater drug-induced dopamine efflux in the nucleus accumbens (93–95). However, a reduction (96) or non-augmentation (97) in the levels of the neurotransmitter in the nucleus accumbens was found simultaneously with this phenomenon. Behavioral sensitization to psychostimulants may well be mediated by converging extracellular signals, which give rise to a number of specific molecular and cellular events, such as activating the ERK/CREB signaling pathway, and enhancing GluR1 AMPA receptor cell surface expression and brain-derived neurotrophic factor/tyrosine kinase B (BDNF/TrkB) receptor signaling within the nucleus accumbens (11, 98). As mentioned previously, the enkephalinergic system increases mesoaccumbal dopamine neurotransmission (25). Likewise, pharmacological studies have demonstrated that MOPr and DOPr receptors contribute to amphetamine (99, 100) and cocaine-induced enhancement of dopamine levels in the nucleus accumbens (84, 86), and there is data of the anatomical selectivity of MOPr receptors within the ventral tegmental area-nucleus accumbens pathway in cocaine-induced reward and locomotor-stimulating effects (101). It has also been proposed that cocaine may cause the release of endogenous opioid peptides. These then activate MOPr within the nucleus accumbens and ventral tegmental area and modulate the drug-induced behavioral effects (102).

The role of MOPr and DOPr in the development and expression of psychostimulant sensitization has been shown pharmacologically. It has been reported that naloxone and naltrexone, non-selective opioid receptor antagonists, attenuate the development of sensitization to cocaine in rats (103) and mice (19, 88, 104). Naltrindole, a DOPr antagonist (87) and CTAP (D-Phe-cyc(Cys-Tyr-D-Trp-Arg-Thr-Pen)-Thr-NH2), a selective MOPr antagonist(105), also reduce cocaine-induced sensitization in rats. Similarly, the development (106) and expression (107, 108) of amphetamine-induced behavioral sensitization were reduced following non-selective opioid receptor administration. Additionally, there is evidence of ERK1/2 signaling stimulation induced by MOPr/DOPr activation in the striatum (109). However, there is data showing that acute morphine caused a reduction in ERK 1/2 levels in the nucleus accumbens (110, 111). Interestingly, although chronic morphine, a MOPr agonist, caused a reduction (110) or tolerance to morphine-induced ERK1/2 activation (111), naloxone-precipitated withdrawal in morphine-dependent animals induced a robust stimulation of ERK1/2 in the striatum (109, 112). Together this evidence demonstrates a prominent role for MOPr in the regulation of molecular events, associated not only with psychostimulant induced-behavioral sensitization, but also with the underlying opiate dependence. However, studies using MOPr mice seem to be inconclusive (88) or did not show a significant influence of this receptor in cocaine sensitization (113–115). It is important to note that these behavioral evaluations were performed after short-term cocaine withdrawal [(88): 10 × 15 mg/kg i.p./7days withdrawal; (113): 5 × 20 mg/kg i.p./dose–response experiment; (114): 6 × 15 mg/kg i.p./6 days withdrawal; (115): 20 mg/kg i.p./3 days withdrawal], possibly masking the role of MOPr in long-term behavioral effects induced by cocaine (116). There is also evidence that, after long- but not short-term withdrawal, naloxone blockade is observed of the expression of behavioral sensitization to psychostimulants [(108): amphetamine 1.5 mg/kg i.p./14 days abstinence]. Despite all these studies, and reports demonstrating that met-enkephalin and MOPr have a prominent role in the ventral tegmental area at the initial step of sensitization (101, 117, 118), there is still no explanation in the literature of the influence of enkephalin on the psychostimulant-induced neuronal plasticity underpinning long-term sensitization. Data from our lab demonstrate an essential role of enkephalin in the development of neuroadaptations in the nucleus accumbens leading to cocaine-induced psychomotor sensitization (19). PENK knockout mice treated chronically with cocaine (9 days x 15 mg/kg) do not become sensitized to cocaine's properties stimulating locomotor activity and dopamine release in the nucleus accumbens 21 days after starting drug treatment. Additionally, the nucleus accumbens and dorsal striatum from PENK knockout mice showed no pivotal neuroadaptations such as the increase in phospho-TrkB receptor, phospho-ERK/CREB and GluR1 AMPA cell surface expression related to sensitized responses to cocaine. Consistent with these observations, full suppression of cocaine-induced behavioral and neuronal plasticity was observed in wild-type animals after naloxone pretreatment (1 mg/kg s.c. 15 min prior to cocaine injections). Reduced activity-dependent BDNF/TrkB signaling within the ventral tegmental area-nucleus accumbens circuit may attenuate the ability of cocaine to induce pathological changes in the nucleus accumbens that promote addiction (119, 120). Related with this, the lack of dopamine sensitization of a cocaine-induced increase in BDNF/TrkB signaling, identified in knockout- and naloxone-pretreated mice, strongly suggests that both enkephalin and BDNF have an important role in dopamine-sensitized behaviors. There is thus considerable evidence that the MOPr/endogenous enkephalin system has a prominent role in the establishment of long-term neuroadaptations within the nucleus accumbens underlying the expression of sensitization to cocaine.

### Conditioned place preference

Pharmacological evidence clearly demonstrates the role of MOPr and DOPr in the modulation of psychostimulant-induced rewarding properties by studying the development of conditioned place preference (CPP); i.e., acquisition of associative learning between a context and the rewarding effects of a drug. In this sense, the establishment of CPP induced by amphetamine was prevented by the non-selective opioid receptor antagonist naloxone (0.02, 0.2 or 2.0 mg/kg s.c.), administered during the conditioning sessions (121). Similarly, naltrexone implants can attenuate cocaine-induced CPP in rats (122), although high doses of the opioid antagonist were required. This effect could be due to the non-selective opioid receptor antagonism. Naltrindole, a highly selective DOPr antagonist, blocked the acquisition of cocaine and amphetamine-induced-CPP in rats (123, 124), indicating that a selective opioid receptor antagonism can fully attenuate the reinforcing properties of cocaine. Furthermore, several studies have demonstrated that selective MOPr receptor antagonists attenuate psychostimulant-induced CPP. Specifically, systemic pretreatment with the selective MOPr type-1 receptor antagonist naloxonazine (125) and intracerebroventricular administration (i.c.v.) of CTAP paired with peripheral injections of cocaine (105), prevented the development of cocaine-induced CPP. It has also been demonstrated that animals pre-treated with CTAP into the nucleus accumbens core or rostral ventral tegmental area, but not into the caudal ventral tegmental area, caudate putamen or medial nucleus accumbens shell, during cocaine conditioning, showed an attenuation of the establishment of cocaine-induced CPP, demonstrating the involvement of mesolimbic MOPr in cocaine-induced reward (101). Although all this evidence has focused on the role of MOPr and DOPr in the development of psychostimulant-induced CPP, their involvement in the expression of this behavior cannot be ruled out. In line, Gerrits et al. (126) assessed the effect of naloxone (0.01–0.1 mg/kg s.c.) administered prior the conditioning test, demonstrating the role of opioid receptors in the expression of cocaine's motivational effects.

Despite this pharmacological evidence, the data regarding cocaine-induced CPP in MOPr knockout mice seems to be inconsistent. For example, the development of cocaine induced-CPP has been reported to be attenuated (113), unchanged (127) or induced after higher doses of cocaine compared to that used in wild-type littermates (128). The mechanisms that underlie these discrepancies in behavioral effects induced by cocaine in MOPr knockout mice are unknown. One possible explanation involves the different protocols of conditioning and cocaine doses used [(128): 4 days conditioning/5 or 10 mg/kg; (127): 3 days–two conditioning sessions per day/10 mg/kg; (113): 2 days–two conditioning sessions per day/5 or 10 mg/kg]. Another explanation could be the genetic background of the mice [(128): 129/Ola × C57BL F2; (113): congenic C57B F10; (127): hybrid 129SV/C57BL/6 F1] that may influence the differences in the process of acquisition of cocaine-induced CPP.

Although the evidence indirectly indicates a potential role of enkephalin in psychostimulant-induced CPP, its role in this process has not been addressed yet. Moreover, the molecular mechanism that underlies the MOPr/DOPr contribution to psychostimulant-induced CPP and the potential role of enkephalin has not been fully studied. Interestingly, there is data suggesting that morphine (a MOPr agonist)-induced CPP is associated with neuroadaptations similar to that observed following chronic psychostimulant treatment in important brain areas associated with drug addiction and those related to memory consolidation. Augmented phosphorylation levels of the GluR1 AMPAR subunit and ERK/CREB were observed in the hippocampus (129–131) as well as in the nucleus accumbens (132) and ventral tegmental area (130, 133) following morphine-induced conditioned behavior. This, together with data from our lab demonstrating that the PENK gene regulates cocaine-induced long-lasting molecular changes, such as enhancement in dopamine transmission, GluR1 AMPA receptor cell surface expression, ERK/CREB signaling pathway activation and modulation of TrkB/BDNF levels in the nucleus accumbens (19), suggests that enkephalin and the MOPr system may favor neuronal plasticity within the mesolimbic circuit that underlies psychostimulant and opiate-induced CPP. Further genetic (PENK knockout mice) and pharmacological studies need to be carried out to confirm this hypothesis and demonstrate the role of enkephalin in psychostimulant-induced CPP.

### Psychostimulant self-administration

There is now considerable pharmacological evidence of the important role that MOPr plays in mediating the reinforcing effects of cocaine in a self-administration paradigm. GSK1521498 (0.1, 1, and 3 mg/kg s.c.), a MOPr antagonist, and naltrexone administered at the same doses and route, reduced cocaine-seeking under a second-order schedule of reinforcement but did not affect cocaine self-administration under a simple fixed-ratio schedule (FR1) (134), indicating modulation of mechanisms regulating cocaine-seeking behavior rather than cocaine reinforcement (135). Additionally, GSK1521498 was more effective than naltrexone in reducing cocaine seeking, possibly because of different opioid receptor subtype selectivity. Similarly, low doses of naltrexone (0.1 mg/kg i.p.) showed no changes in cocaine self-administration (FR2 schedule), but attenuated cocaine- and cue-induced reinstatement of drug-seeking behavior administered 30 min prior to the reinstatement test (136). Consistently, the MOPr irreversible antagonist, beta-funaltrexamine, administered intra-ventral tegmental area or nucleus accumbens, had no effect on cocaine self-administration under a FR1 schedule of reinforcement. In contrast, MOPr blockade in both brain regions did attenuate the response to cocaine under a progressive ratio (PR) schedule, supporting the notion that MOPr within the mesolimbic system is involved in motivation to respond to cocaine (137). Regarding the role of MOPr, the selective MOPr antagonist CTAP (0.3 and 3 μg) administered in the ventral pallidum, but not in the nucleus accumbens or lateral hypothalamus, blocked the reinstatement of drug-seeking in rats that extinguished from cocaine self-administration (138). Given the GABA/enkephalin projection from the nucleus accumbens to the ventral pallidum, chronic cocaine may result in enkephalin release in this brain area, activating MOPr and eliciting cocaine relapse.

Data regarding the role of DOPr in mediating the rewarding effects of cocaine are conflicting. Naltrindole (0.03–3.0 mg/kg i.p. prior to self-administration session) did not alter the intake of cocaine (FR2 schedule of reinforcement) or the re-acquisition of cocaine self-administration (139). Similarly, a selective DOPr type-2 antagonist (administered i.c.v.) has been reported to have a slight effect on cocaine self-administration (FR1 schedule) (140). In contrast, there is data demonstrating that naltrindole (10 mg/kg i.p. 15 min prior FR1) reduced cocaine self-administration (141). These discrepancies regarding the role of DOPr in cocaine reinforcement may be due to the different types and doses of DOPr antagonist and cocaine-self-administration protocols. Importantly, none of these studies evaluated a possible role of DOPr within specific brain areas associated with cocaine reinforcement. DAMGO (1–3 ng) and DPDPE (300–3,000 ng), MOPr- and DOPr-selective ligands respectively, as well as β-endorphin (100–1,000 ng) and the enkephalinase inhibitor thiorphan (3–10 μg) microinjected into the nucleus accumbens, are sufficient to reinstate cocaine-seeking behavior in rats following extinction of cocaine self-administration (142). Thus, the stimulation of either accumbal MOPr or DOPr seems to be necessary to precipitate cocaine relapse.

Cocaine self-administration was reduced in MOPr knockout mice (143), suggesting a critical role of this receptor in cocaine reinforcement. In contrast, Gutiérrez-Cuesta et al. (144), found no changes in cocaine self-administration in this genotype. This discrepancy could be explained in the framework of the differences in experimental protocols used regarding cocaine dose and the time of the conditioning sessions, as in the study of Mathon et al. (143), which demonstrated significant differences in this genotype at high cocaine doses in shorter session times. Moreover, cocaine self-administration was reduced in both DOPr knockout and PENK knockout mice (144), mainly when animals were trained in FR3 and PR schedules. These findings suggest that DOPr and PENK are involved in the motivation to obtain cocaine, and the absence of these opioid components engenders an impaired response of cocaine self-administration, mainly when greater effort to obtain a reward is required. In addition, Gutiérrez-Cuesta et al. (144), demonstrated that cue-induced reinstatement of cocaine-seeking behavior was attenuated in both DOPr knockout and MOPr knockout. These data support previous pharmacological studies of Simmons and Self (142) addressing an important role of both receptors within the mesolimbic system in cocaine relapse. Consistent with these data, an enduring MOPr tone has been demonstrated within brain reward structures following extinction of cocaine self-administration (145), indicating that up regulating enkephalin levels may lead to long-lasting adaptations in response to repeated cocaine. Thus, all this evidence indicates that enkephalin, presumably acting on MOPr (although a role of DOPr cannot be ruled out) has a facilitatory influence on cocaine-induced behavioral and neuronal plasticity.

Importantly, the human literature shows encouraging evidence regarding the use of opioid antagonist in the treatment of psychostimulants relapse (146–148). Indeed, naltrexone (50 mg/day) administered in combination with relapse prevention therapy reduced cocaine use in a study of cocaine-addicted patients (*n* = 85). Thus, people receiving the combination of naltrexone (administered throughout 12 weeks) and relapse prevention therapy evidenced significantly reduced cocaine use than participants receiving other treatment combinations such us naltrexone alone or combined with drug counseling therapy (147). The same treatment protocol (naltrexone 50 mg/day during 12 weeks of medication and relapse prevention therapy) reduced amphetamine use as well as craving in amphetamine dependent patients (*n* = 55) (149). Additionally, naltrexone (50 mg/day) reduced the subjective effects of dexamphetamine (30 mg, oral) in amphetamine-dependent people (*n* = 20) (150). In constrast, patients who received oral naltrexone doses (0, 12.5, or 50 mg) before smoked cocaine (0, 12.5, 25, and 50 mg or placebo), or oral amphetamine (0, 10, and 20 mg or placebo) did not show alterations in positive subjective effects in cocaine users (*n* = 12) (146). This evidence suggests that this opioid antagonist did not alter positive subjective ratings after cocaine. Importantly, naltrexone did not alter physiological effects of psychostimulants in terms of cardiovascular function (146), cortisol levels and skin conductance (149, 150). Morever, naltrexone did significantly reduce craving for cocaine and tobacco during cocaine sessions (146) as well as amphetamine craving (149, 150). These data demonstrated that behavioral alterations observed in psychostimulants addiction, such us drug craving could be modulated by the endogenous opioid system.

It is important to address that in these studies, participants do not show evidence of any increase in the intake of other drugs of abuse during naltrexone protocol therapy to compensate for the reduction in the drug consumption that is being evaluated. On the other hand, these studies were restricted to short periods of naltrexone treatment and long-term effects in these patients are unknown. Thus, future longitudinal studies are required in order to follow patients over prolonged periods of time.

Similar effects on opioid antagonists were observed in patients with cocaine/alcohol comorbidity (148, 151–153) or cocaine/opiate dependence (154).

In summary, several studies show promising results for psychostimulants addiction treatment, suggesting a potential role of naltrexone as an anti-craving therapy for this psychiatric disorder.

## Conclusions and future directions

This review emphasizes the important role of endogenous enkephalin during the development of the long-term neurobiological changes underlying psychostimulant addiction. It has been suggested that polymorphisms in genes encoding components of the endogenous opioid system are involved in predisposing to addiction to cocaine and opiates (155). Similarly, it is likely that genetic variations in the endogenous PENK gene (155–158) influence the development of behavioral and neurobiological adaptations in response to psychostimulant exposure, and thus modify vulnerability to psychostimulant addiction. This review also helps to understand how opioid antagonists can be effective in treating psychostimulant addiction (146, 147, 149), supporting their use as therapy for this disorder. Thus, the evidence presented in this review provides a basis for the development of new drug therapies for psychostimulant addiction based on specific modulation of the endogenous PENK system.

## Author contributions

BMB wrote the article and performed the research related with the topic of this review; MPA, ASG and FAB contributed with the research related with the topic of this review and the writing of the review; LMC wrote the article, designed the research and provided the funds to perform papers related with the topic of this review.

### Conflict of interest statement

The authors declare that the research was conducted in the absence of any commercial or financial relationships that could be construed as a potential conflict of interest.
